# Low Exercise Tolerance as a Marker of Erectile Dysfunction and Depression among Post-Myocardial Infarction Men

**DOI:** 10.3390/healthcare11091213

**Published:** 2023-04-24

**Authors:** Amanda Mandera-Grygierzec, Paulina Kostrzewska, Ewa Szuster, Anna Pawlikowska-Gorzelańczyk, Małgorzata Biernikiewicz, Agnieszka Rusiecka, Aneta Mrozek-Szetela, Małgorzata Sobieszczańska, Krystyna Rożek-Piechura, Monika Markiewicz, Dariusz Kałka

**Affiliations:** 1 Cardiosexology Students Club, Wroclaw Medical University, 50-368 Wroclaw, Poland; 2 Studio Słowa, 50-357 Wrocław, Poland; 3 Statistical Analysis Centre, Wroclaw Medical University, 50-367 Wrocław, Poland; 4 Doctoral School at Wroclaw of Environmental and Life Science, 50-375 Wrocław, Poland; 5 Clinical Department of Geriatrics, Wroclaw Medical University, 50-369 Wrocław, Poland; 6 Faculty of Physiotherapy, Wroclaw University of Health and Sport Sciences, 51-612 Wrocław, Poland; 7 Men’s Health Centre in Wroclaw, 53-151 Wrocław, Poland

**Keywords:** sexual dysfunction, erectile dysfunction, men’s health, physical activity, mental health, Polish population

## Abstract

Evidence has grown recently on the correlation between lifestyle and physical activity, and their impact on the functioning of the entire organism. In addition, a decrease in physical efficiency may be an indicator of the early diagnosis of systemic diseases. The aim of this study was to determine whether there is a relationship between exercise tolerance and possible erectile dysfunction or mental disorders. A cross-sectional study was conducted among 254 men in 4 cardiac rehabilitation centers in Poland using the standardized International Index of Erectile Function 5 (IIEF-5) and Beck Depression Inventory (BDI) questionnaires. Erectile dysfunction was directly proportional to the metabolic equivalent of the task (MET) variable. An increase in exercise tolerance by 1 point was associated with an increase in the IIEF-5 score by 1.62 points, indicating a reduction in the severity of erectile dysfunction. The 1-point increase in MET was associated with a 1.8-point decrease in BDI, indicating that an increase in exercise tolerance is associated with a decrease in the severity of depressive disorders. Increasing the tolerance of physical effort has a beneficial effect not only on the general well-being, but also on the sexual and mental health of men. An increase in exercise tolerance is associated with a reduction in the incidence of erectile dysfunction. On the other hand, in patients with depressive disorders, the improvement of exercise tolerance reduces the intensity of depression symptoms. Exercise tolerance can be an early and simple marker at the onset of erectile dysfunction or mood disorders.

## 1. Introduction

Sexual dysfunction is a group of sexual behavior and experience disorders that appear as abnormal or missing physiological responses. It is a term that includes the definition of erectile dysfunction (ED), the failure of sexual intercourse or the loss of desire/libido. Male sexuality is actually a component of several factors and is a significant part of every man’s life. Maintaining proper sexual function depends on the interaction of the nervous, cardiovascular, endocrine and reproductive systems [1,2].

Disorders in the work of any system negatively affect the proper functions of sexual life. Sexual dysfunction is not a single disease; it is a collection of various male sexual activities leading to a reduction in quality of life. In 1992, scientists at the National Institutes of Health defined the concept of ED as the inability to achieve or maintain adequate penis stiffness to complete sexual intercourse [3].

Sexual dysfunction is common in all age groups of men, regardless of their ethnic or cultural background. According to a study by the Massachusetts Male Aging Study, more than 50% of men aged 40 to 70 years suffer from varying degrees of sexual dysfunction [4]. In men aged 40 to 49 years, disorders occur in 1–29%, while this value increases significantly in men over 70 years of age and reaches values between 26–76% [5]. Over 1.5 million men over 35 years of age suffer from ED in Poland and only 15% of them seek medical advice [6].

Chronic disease-induced impotence is a growing problem. The available literature includes numerous studies showing the influence of cardiovascular disease, diabetes, arterial pressure, and hyperlipidemia on the development of sexual disorders [7,8,9,10]. In addition, lifestyle diseases, which are largely influenced by a lack of physical activity, become more prevalent [11].

ED significantly deteriorates a man’s quality of life. Until recently, it has been believed that in most cases these disorders were psychogenic. Currently, on the basis of numerous studies, it has been proven that over 80% of cases have an organic etiology-endocrine or non-endocrine [12]. Often, organic ED manifests itself in a psychological form. Patients suffering from ED mainly show symptoms of depression and anxiety disorders related to impaired sexual performance. This, in turn, directly affects a couple’s quality of life, interpersonal relationships and their mood.

ED and cardiovascular disease share many risk factors such as age, smoking, excessive alcohol consumption, a high body mass index (BMI), and a sedentary lifestyle [13].

Importantly, ED is not limited to sexual activity, but is a very good indicator and predictor of systemic endothelial dysfunction [14]. From a practical point of view, ED very often precedes cardiovascular events and can be an early marker for identifying patients at high risk of cardiovascular disease [12].

Changing lifestyle, increasing physical activity, and maintaining healthy body weight have a preventive and protective effect against ED and may be more likely to be effective than ED treatment [15]. Physical activity is also very important in the treatment of depression.

An MET (Metabolic Equivalent of Task) is an important, standardized parameter that enables the comparison of physical activity in people of different body weight and those undertaking various activities. It is a unit of energy used by the body during physical activity in relation to resting metabolism. The MET is a parameter that determines the metabolic rate expressed as the amount of oxygen consumed at rest (sitting in a chair), which is equal to approximately 3.5 mL O_2_/kg/min (12 kcal/min for a person weighing 70 kg). A person at rest consumes an average of 1 MET, which corresponds to 1 kcal/kg of body weight for active minutes, and 3.5 milliliters of oxygen per kilogram of body weight multiplied by the minutes of activity. An acceptable level of physical activity, which can be considered as general physical well-being, can be defined as practicing physical activity with an intensity of 2.5 MET/min for 30 min, 5 times a week, i.e., 375 MET/min per week [16].

Approximately 280 million people in the world have depression, including 5.0% of adults and 5.7% of adults older than 60 years [17]. Depression is a disorder of the emotional life, which the main symptom is a dominant feeling of sadness, despondency, and discouragement [18]. It is a disease that disrupts the functioning of both the family and professional spheres. In the most severe cases, depression can lead to suicide. Each year, 700,000 people take their own lives because of depression. Research shows a number of interrelationships between depression and physical health. Prevention programs based on physical exercise have also been shown to bring very good results [17].

The aim of our study was to investigate the correlation between risk factors for depression, erectile dysfunction and exercise tolerance.

## 2. Materials and Methods

The cross-sectional study was conducted among 254 men in 4 cardiac rehabilitation centers in Poland. The standardized International Index of Erectile Function 5 (IIEF-5) and the Beck Depression Inventory (BDI) questionnaires were used to conduct the study. The questionnaires included additional sociodemographic and clinical questions. The participants were asked to complete questionnaires and were informed of voluntary and anonymous participation in the survey. The study was approved by the Commission of Bioethics at Wroclaw Medical University, Wrocław, Poland (No. KB-433/2010). The study was part of the PREVANDRO project.

The IIEF-5 is a self-administered sexual activity questionnaire to assess erectile dysfunction. Each answer is scored on a scale of 0–5. The survey consists of 5 questions that concern erectile function and intercourse satisfaction with the partner. The total analyzed score ranges from 5 to 25 points. The severity of ED was classified as: severe (5–7); moderate (8–11); mild to moderate (12–16); mild (17–21); and without ED (22–25) [19].

BDI is a self-report inventory composed of 21 multiple-choice questions to measure the severity of depression on a four-point scale (0–3) [20]. Higher total scores signify more severe depressive symptoms. The standard cut-off scores are: 0–11 minimal depression; 12–19 mild depression; 20–25 moderate depression; and 26–63 severe depression.

The metabolic equivalent of task (MET) is the oxygen demand of various activities that increases with the intensity of physical exertion. One MET is the amount of oxygen consumed while seated in a resting position, which is approximately 3.5 mL O2/kg/min [21]. The resting metabolic rate is considered to be independent of body weight and therefore relatively constant for all persons [22]. The caloric cost of physical activity can be estimated from the equation: kcal = MET × weight in kilograms × duration in hours [23].

The questionnaire enabled the collection of demographic data and chronic diseases, such as hypertension, dyslipidemia, diabetes, and obesity. The interview also included questions about smoking and clinical data collected during the qualification for cardiac rehabilitation.

Statistica software v.13.1 (StatSoft, Tulsa, OK, USA) was used for data analysis. These data were presented as numbers, percentages, and means with standard deviations. The Shapiro–Wilk test was used to analyze the distribution of these data. The Chi square test was used for the comparison of qualitative variables. For comparisons between groups of variables with a normal distribution, Student’s t-test for independent variables was used. The differences were considered statistically significant at a *p*-value <0.05.

## 3. Results

The study group consisted of 254 men who met the criteria and completed questionnaires. The mean age of the respondents was 56.54 ± 5.47 years. The clinical characteristics of the study group are presented in Table 1 and Table 2.

Among the risk factors examined in the study, a significant correlation between ED and smoking in the past was noted (*p* = 0.021). Moreover, we found that diabetes mellitus is a factor that has a significant impact on the occurrence of depression (*p* = 0.00001). However, we did not find a statistically significant impact of the other mentioned risk factors for ED. The detailed results of the impact that risk factors have on depressive disorders are presented in Table 3.

However, we did not find a statistically significant impact of the mentioned risk factors for ED. The correlations between the analyzed risk factors and ED are presented in Table 4.

**Figure 1:**
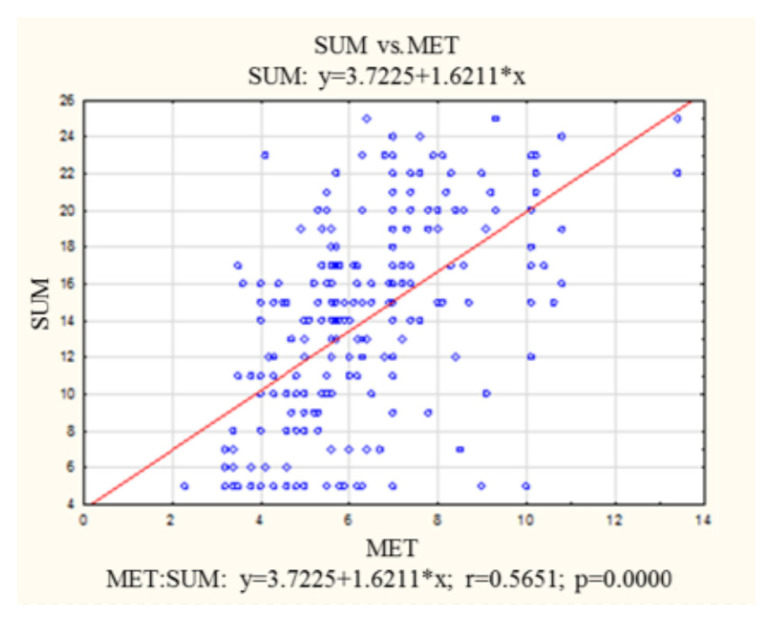
Correlation between IIEF-5 score and metabolic equivalent of task. MET—metabolic equivalent of task; IIEF-5—International Index of Erectile Function 5.

ED depends on MET in direct proportion. An increase in exercise tolerance by 1 point is associated with an increase in the IIEF-5 result by 1.62 points, which indicates a reduction in the severity of ED. The correlation between the IIEF-5 score and MET is presented in Figure 1.

MET scores varied significantly with the presence or absence of a depressive disorder. The axis of abscissa in these groups is approximately 9 BDI points. Depressive disorders were not observed in patients who achieved at least 7 MET points.

**Figure 2:**
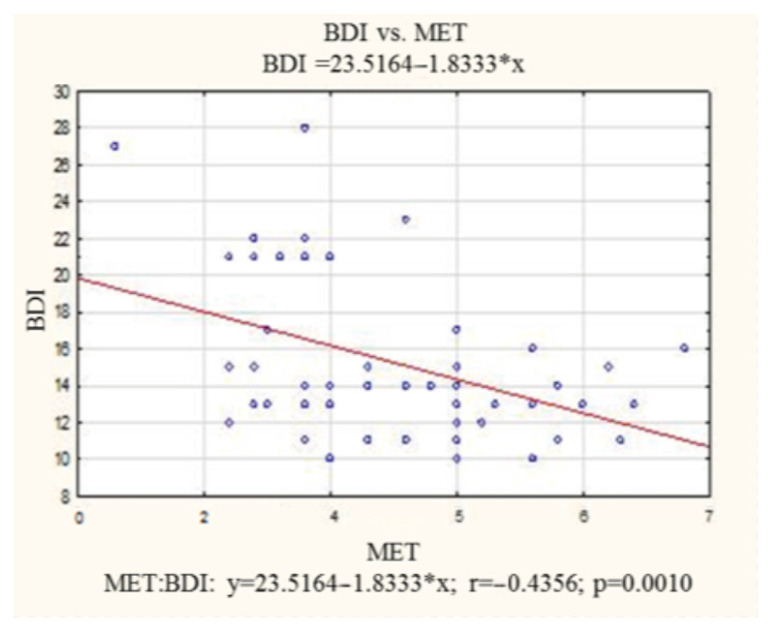
Correlation between depressive disorder and metabolic equivalent of task among patient with depression. BDI—Beck Depression Inventory; MET—metabolic equivalent of task.

There was a statistically significant negative correlation between exercise tolerance expressed in MET and the severity of depressive disorders (r = −0.43) among patients with depression. The increase in MET by 1 point was related to the decrease in the BDI by 1.8 points, indicating that an increase in exercise tolerance is associated with a decrease in the severity of depressive disorders. The correlation between depressive disorders and MET is presented in Figure 2.

**Figure 3:**
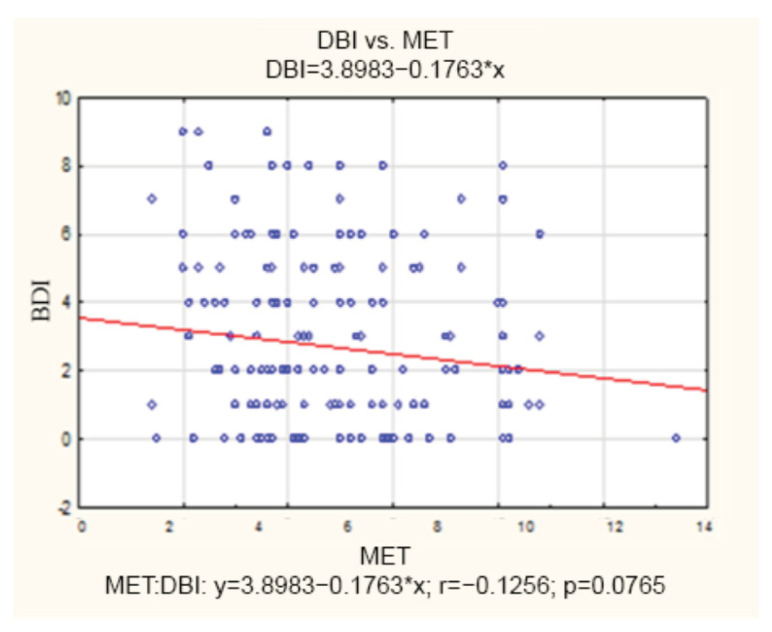
Correlation between the Beck Depression Inventory and metabolic equivalent of task among patients without depression. BDI—Beck Depression Inventory; MET—metabolic equivalent of task.

There was no correlation between the BDI and MET scores among patients without depression. The correlation coefficient was not statistically significant (r = −0.12). The 1-point increase in MET was related to the 0.2-point decrease in BDI. The detailed data are presented in Figure 3.

## 4. Discussion

Identifying pathogenetic factors is essential for an accurate diagnosis and effective treatment of ED [24]. Factors that significantly increase the risk of ED are diabetes, hypertension, dyslipidemia and depression [25]. Additionally, the influence of cigarette smoking, obesity, sedentary lifestyle and chronic alcohol use on the occurrence of ED is noticeable [26,27]. Modifiable lifestyle factors are involved in the occurrence of ED, which indicates the role of behavior modification in the prevention of ED [16,28].

The analysis of the results confirmed the positive effect of better exercise tolerance on the severity of ED. These results are consistent with many other previous cross-sectional studies examining the association between ED and physical activity [28,29,30]. Bacon et al. showed that frequent strenuous exercise was associated with a lower risk of ED than much less or no exercise. Furthermore, men under 60 years of age have been shown to benefit more from exercise than older men (80 years of age) [30]. Physical exercise resulted in increased testosterone levels, which may explain the correlation between physical activity and male sexual function [31]. Reducing excess body weight in obese men through increased physical activity also led to an improvement in sexual function [32].

Paulsen et al. conducted a study among nearly 50,000 Danish men and confirmed statistically significant relationships between the self-assessment of physical fitness and reported ED [33]. The worse the patients assessed their exercise tolerance, the more often they reported sexual dysfunction.

The vascular endothelium is a single layer of cells that forms a biologic interface between circulating blood elements and the various systems in the body. It plays a vital physiological role in vascular homeostasis by synthesizing and releasing a number of biologically active factors involved in the regulation of vascular tone, platelet aggregation, monocyte and leucocyte adhesion, thrombosis and smooth muscle [34].

There is increasing evidence in the available literature that endothelial dysfunction is one of the earliest signals in the pathogenesis of cardiovascular diseases, erectile dysfunction and brain diseases. Undertaking regular physical activity may be a non-pharmacological therapeutic option to delay the decline in endothelial function associated with aging. Regular participation in endurance exercise may attenuate the ageing-related decline in endothelial function [35].

Mental health is another integral part of health. There are many studies around the world that try to identify the risk factors associated with depression. Researchers revealed that ex-smokers, as well as current smokers, are at an increased risk of depression [36,37]. Other risk factors for depression include lower childhood socioeconomic status [38], a number of illnesses [36], low HDL-cholesterol levels [39] and hypertension [40]. However, physical activity was mentioned as a protective factor [41]. Our study has also revealed that better exercise tolerance was correlated with a lower severity of depressive disorders. In addition, there is also a correlation between BDI and smoking in the past. As in the above-mentioned studies, we found no link between BDI and BMI. It is important to notice and implement effective interventions to prevent depression by modifying established risk factors. Numerous studies show that physical activity can prevent future depression [42,43].

In addition, physical activity itself may reduce the occurrence of depressive symptoms, and thus reduce the risk of ED. The study by Kim et al. showed the preventive effect of optimal physical activity on the occurrence of depressive disorders [44]. Based on the results of the Southern California community survey, a positive correlation was found between BDI and the level of free testosterone. Furthermore, free testosterone concentration was inversely correlated with age, lack of regular physical activity, and weight loss [45].

The study by Ma et al. confirmed earlier assumptions that depression increases the incidence of ED at the genetic level [46]. As early as 2000, Goldstein explained the biological hypothesis of ED [47]. Both ED and the abnormal relaxation of the cavernous muscles of the penis were found to be caused by the overproduction of catecholamines, which was influenced by depression. In addition, the dopamine system and the dopaminergic synapse signaling pathway were abnormal in the rat model of depression [48].

The appearance of ED may lead to the development of depressive disorders; therefore, it is important that patients with sexual dysfunction are screened for depression [49].

Our findings are an important clue for physicians of many specialties, who in their daily practice encounter a common problem of reduced exercise tolerance. It is especially important to collect the correct medical history and to conduct an initial analysis of ED and depression as early as possible. This would have a significant impact on the early detection of disorders that, in the long run, reduce quality of life.

Several methodological limitations of this survey should be considered. The study was conducted among patients with coronary artery disease, so it is not representative of the entire male population. Moreover, both the BDI and IIEF-5 scales used in the study are self-assessment tools, which can lead to subjective responses and recall bias. In addition, sexual activity also depends on the sexual needs of the partner, which was not included in the research. In this study, we focused on the correlation between sexual dysfunction and exercise tolerance as risk factors for depression. It should be remembered that prioritization ignores many factors that may also affect ED, and therefore they may be taken into account in future research.

## 5. Conclusions

The study showed that exercise tolerance is closely related to ED and depression, and is an early and simple marker of these conditions. A high score of exercise tolerance has a positive effect on the sexual health and mental state of men. The increase in exercise tolerance is associated with a decrease in the incidence of ED. In patients with depression, the increase in exercise tolerance is associated with a decrease in the severity of depressive disorders.

## Figures and Tables

**Table 1:** Clinical characteristics of the study group.

Clinical Data	Study Group *n* (%)
Height (m)	1.73 ± 0.06
Weight (kg)	84.36 ± 12.44
BMI (kg/m^2^)	28.10 ± 3.73
Waist circumference (cm)	98.56 ± 11.51
Overweight and obesity	203 (79.92%)
Hypertension	54 (21.26%)
Diabetes type 2	103 (40.55%)
Lipid disorders	149 (58.66%)
Smoking now	14 (5.51%)
Smoking in the past	208 (81.89%)
Cigarettes per day	20.16 ± 8.38
Pack years	46.09 ± 21.43
Sedentary lifestyle	223 (87.80%)
LTPA +	31 (12.20%)
LTPA (any activity)	117 (87.80%)
Mean LTPA (kcal/week)	798.13 ± 403.98
Creatinine	1.04 ± 0.53
HGB	13.1 ± 1.67
Potassium	4.31 ± 0.39
Glucose	108.1 ± 34.13
CHOL	148 ± 38.63
TRIGL	123.5 ± 65.29
HDL	42.16 ± 10.34
LDL	81.1 ± 30.05
Atrial fibrillation	38 (14.96%)
Myocardial infarction	254 (100%)
PTCA	170 (68.55%)
CABG	115 (46.37%)

BMI, body mass index; LTPA, Leisure-time physical activity; HR, heart rate; HRR, heart rate recovery; HGB, hemoglobin; CHOL, total cholesterol; TRIGL, triglycerides; HDL, high-density lipoprotein, LDL, low-density lipoprotein; TRIGL, triglycerides; PTCA, percutaneous transluminal coronary angioplasty; CABG, coronary artery bypass graft.

**Table 2:** MET before and after rehabilitation.

Clinical Data	Study Group *n* (%)
MET before rehabilitation	6.39 ± 1.94
MET after rehabilitation	7.43 ± 2.16

MET, metabolic equivalent of task.

**Table 3:** Risk factors for ED in the study group.

		*n*	Depression *	No Depression *	*p*-Value	ED	Non-ED	*p*-Value
BMI	≥25	203	43	160	0.96	103	20	0.99
	<25	51	11	40		46	5	
Lipid disorders	yes	149	31	118	0.83	133	16	0.57
	no	105	23	82		96	9	
Smoking in the past	yes	208	50	158	0.02	190	18	0.18
	no	46	4	42		39	7	
Smoking now	yes	14	2	12	0.18	12	2	0.77
	no	240	23	217		217	23	
Hypertension	yes	200	43	157	0.86	177	23	0.35
	no	54	11	43		52	2	
Diabetes mellitus	yes	103	36	67	0.00001	98	5	0.03
	no	151	18	133				

* According to the BDI questionnaire. BDI—Beck Depression Inventory; BMI—body mass index; ED—erectile dysfunction; IIEF-5—International Index of Erectile Function 5.

**Table 4:** Correlation between the occurrence of risk factors for ED in the study group.

		*n*	ED	Non-ED	*p*-Value
BMI	≥25	203	103	20	0.99
	<25	51	46	5	
Lipid disorders	yes	149	133	16	0.57
	no	105	96	9	
Smoking in the past	yes	208	190	18	0.18
	no	46	39	7	
Smoking now	yes	14	12	2	0.77
	no	240	217	23	
Hypertension	yes	200	177	23	0.35
	no	54	52	2	
Diabetes mellitus	yes	103	98	5	0.03
	no	151	131	20	

According to the IIEF-5 questionnaire. BMI—body mass index; ED—erectile dysfunction; IIEF-5—International Index of Erectile Function.

## Data Availability

Not applicable.
